# An update to the global Critical Habitat screening layer

**DOI:** 10.1038/s41597-025-06117-y

**Published:** 2025-11-18

**Authors:** Sebastian Dunnett, Alfred Muge, Alex Ross, Joseph A. Turner, Neil D. Burgess, Matt Jones, Sharon Brooks

**Affiliations:** 1 UN Environment Programme World Conservation Monitoring Centre, Cambridge, CB3 0DL UK; 2 Bluedot Associates Ltd, 12 Whiteladies Rd, Clifton, Bristol BS8 1PD UK

**Keywords:** Business, Biodiversity, Conservation biology

## Abstract

The International Finance Corporation (IFC) defines Critical Habitat in Performance Standard 6 (PS6) as high biodiversity value areas requiring net biodiversity gain for projects. We present an updated global screening layer of Critical Habitat aligned with IFC’s 2019 guidance. This layer derives from global datasets covering 54 biodiversity features, categorized as ‘Likely’ or ‘Potential’ Critical Habitat based on alignment with IFC criteria and data suitability. Analysis indicates 53.95 million km^2^ (10.58%) and 13.71 million km^2^ (2.69%) of the globe can be considered Likely and Potential Critical Habitat respectively, with the remaining 86.73% not overlapping with assessed biodiversity features. This represents a significant increase over previous efforts but likely remains a significant underestimation of actual Critical Habitat. Likely Critical Habitat was dominated by Important Bird and Biodiversity Areas, Intact Forest Landscapes, and protected areas; Potential Critical Habitat by Important Marine Mammal Areas and ranges of IUCN Vulnerable species.

## Background & Summary

More than US$ 60 trillion of infrastructure spending is likely required to meet 2040 societal goals, with an additional 1.2 million km^2^ urbanised land by 2030, an additional 3–4.7 million km of roads by 2050^1–3^. Despite the relatively modest infrastructure expansion of the late twentieth and early twenty-first centuries, biodiversity has continued to decline precipitously, with governments collectively failing every single one of the Aichi Biodiversity Targets set under the United Nations (UN) Convention on Biological Diversity (CBD) in 2011. Much of the potential infrastructure is predicted to be newly built in some of the world’s highest integrity ecosystems, potentially leading to even greater declines in biodiversity globally^4–7^.

In 2012, the International Finance Corporation (IFC; a member of the World Bank Group) revised their *Performance Standard 6: Biodiversity Conservation and Sustainable Management of Living Natural Resources* (hereafter PS6), one of eight Performance Standards any client of the organisation must meet throughout the life of an investment^8^. PS6 draws heavily on fundamental conservation principles such as protected areas and threatened species^9–11^. The standard is seen as the benchmark for sustainable investment with 130 financial institutions following its social and environmental classification process as signatories to the Equator Principles^12^. Businesses can prioritise impact avoidance and direct corporate nature action with Critical Habitat screening^13^, even more important when, for example, half of marine Critical Habitat identified in 2015 is not formally protected and would not be flagged when only considering legally designated sites^14^.

Critical Habitat is defined by IFC using the following criteria:

1. Critically Endangered or Endangered species
2. Endemic and/or restricted-range species
3. Globally significant concentration of migratory or congregatory species
4. Highly threatened and/or unique ecosystems
5. Key evolutionary processes

Previous work by Martin *et al*.^15^ and Brauneder *et al*.^16^ produced global 1 km^2^ IFC Critical Habitat screening layers for the marine and terrestrial realms respectively. These studies identified global biodiversity feature data with sufficient resolution and alignment with IFC PS6 criteria to produce global screening layers for Likely and Potential Critical Habitat. Martin *et al*. classified 1.6% of the ocean as Likely Critical Habitat and 2.1% as Potential Critical Habitat. Brauneder *et al*. 16 identified 10% of the terrestrial surface as Likely Critical Habitat and 5% as Potential Critical Habitat. The screening layers, as well as the amalgamated marine plus terrestrial Critical Habitat layer, are made available through the UN Environment Programme World Conservation Monitoring Centre’s data portal (https://data-gis.unep-wcmc.org/portal/home/) as well as the UN Biodiversity Lab (https://unbiodiversitylab.org/en/). The layers have been used in a number of subsequent studies, including analysis of the impact of PS6 on biodiversity offset implementation^17^. Many of the studies focus on the threat to biodiversity of China’s Belt and Road Initiative, a programme to link sixty-five countries with a network of transport and energy infrastructure. Researchers have used the global Critical Habitat screening layer to estimate that 50% of loans intersect with Critical Habitat^18^. Reference to Critical Habitat in this manuscript refers specifically to Likely or Potential Critical Habitat. This **does not** indicate confirmed Critical Habitat, which requires ground-truthed assessment. In total, 49 Chinese-funded dams have 149 km^2^ Critical Habitat in close proximity (i.e. within 1 km), whereas dams funded by multilateral development banks (MDBs), with binding biodiversity safeguards, have much lower average areas of Critical Habitat per area of dam at risk^19^. Researchers have also identified 12,000 km^2^ of Critical Habitat within 1 km of the Belt and Road Initiative’s linear infrastructure^20^. In a decarbonising world where demand for minerals is likely to increase, 20% of mining locations in Africa trigger a Critical Habitat classification for something other than Great Ape habitat^21^.

In June 2019, IFC updated the 2012 PS6 Guidance Note that had accompanied the initial release^22^. The update significantly changed the Critical Habitat criteria to better align the standard with the then newly developed Global Standard for the Identification of Key Biodiversity Areas (KBAs), Table 1. This meant that the original Critical Habitat global screening layers were now out of out of data with respect to the latest criteria. At the same time, many of the datasets that underpinned the original analyses have been updated multiple times (e.g. the World Database on Protected Areas – WDPA – is updated monthly). Finally, many new datasets have been produced over recent years that meet the completeness and alignment criteria of the layer.

**Table 1:** Comparison of thresholds for triggering Critical Habitat in the update Guidance Note.

Criteria	GN2012	GN2019	Implications
Critically Endangered or Endangered species	Tier 1: 10% Tier 2: > 0%	0.5%	Small increase in threshold Reduction in overall Likely or Potential Critical Habitat
Endemic and/or restricted-range species	Tier 1: 95% Tier 2: 1%	10%	Threshold significantly higher Reduction in overall Likely or Potential Critical Habitat
Globally significant concentrations of migratory or congregatory species	Tier 1: 95% Tier 2: 1%	1%	Threshold unchanged No change to overall Likely or Potential Critical Habitat
Highly threatened and/or unique ecosystems	Qualitative No thresholds	5%	New threshold
Key evolutionary processes	Qualitative No threshold	Qualitative No threshold	

Implications are based on changes to the lowest threshold for Critical Habitat (i.e. Tier 2 in 2012). Adapted from UNEP-WCMC^32^.

For businesses to manage their biodiversity impacts in areas of high biodiversity value, an updated Critical Habitat layer is required that fully aligns with the 2019 update of PS6. In this Data Descriptor, we present such a layer, which incorporates updated datasets, as well as new data sources identified through a comprehensive search against the eligibility criteria. We also describe and make available a methodology whereby the layer can be updated as and when new data are found or updated.

## Methods

### Critical Habitat criteria

IFC define Critical Habitat according to five criteria (Table 1) with associated thresholds for Criteria 1-4. The 2019 update to the accompanying Guidance Note made several changes to the classification of Critical Habitat of relevance to any updated methodology:

- **Changes to thresholds**: thresholds have been updated to better align with the Global KBA Standard^23^. Criteria 1, 3 and 4 now directly align with the standard, and Criteria 1-3 have streamlined thresholds that largely sit between the previous Tier 1 and Tier 2 thresholds. Quantitative thresholds have been added for Criterion 4, based on the developing IUCN Red List of Ecosystems^24^.
- **Specified exceptional circumstances**: IFC now specify three circumstances that trigger special considerations.
  - Great Apes: the presence of Great Ape species is likely to trigger Critical Habitat regardless of thresholds. Where present, clients are expected to inform both IFC and the IUCN Species Survival Commission’s Primate Specialist Group.
  - Unapprovable sites:
    - World Heritage Sites (Natural or Mixed).
    - Alliance for Zero Extinction sites.
- **Addition of IUCN Vulnerable species**: IFC added a threshold for Criteria 1 that now explicitly includes species classified as Vulnerable (VU) by the IUCN Red List of Threatened Species (hereafter IUCN Red List) where:
  - Globally important populations are present.
  - The loss of the population would lead to the species being upgraded to Endangered (EN) or Critically Endangered (CR).
  - The population in question would then trigger Criterion 1a (the area supports >  = 0.5% of the global population and >= 5 reproductive units).

### Data screening and classification

Using the updated criteria, we proceeded with data screening and classification as with previous studies^15,16^. Datasets were identified through expert knowledge and consultation, using the criteria adapted from Martin *et al*.:

1. Direct relevance to one or more Critical Habitat criteria.
2. Global in extent.
3. Assembled using a standardised protocol.
4. The best available data for the biodiversity feature of interest.
5. Sufficiently high resolution to indicate presence of biodiversity on the ground at scales relevant to business operations.

**Fig. 1:**
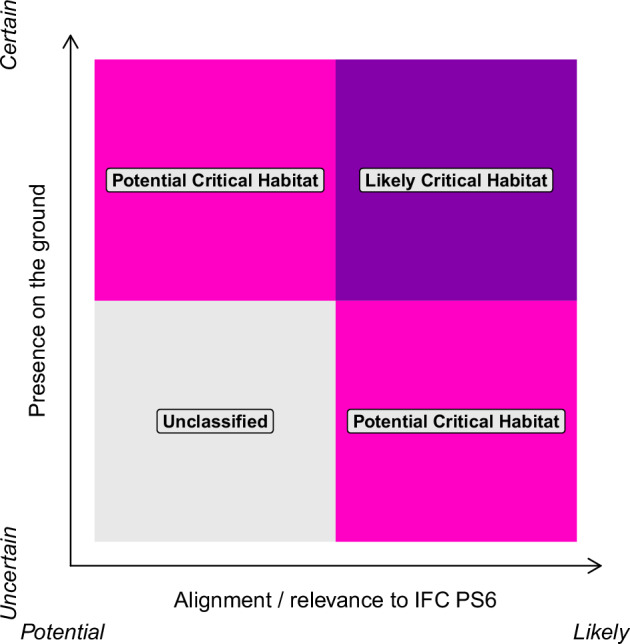
Classification of data as Likely or Potential Critical Habitat is based on the perceived strength of alignment with PS6 criteria and the spatial resolution of the data. Adapted from Brauneder *et al*.^16^.

Datasets that met our criteria were classified as one of three classes: Likely, Potential, and Unclassified. The classification was done by expert judgement, weighing the alignment with PS6 criteria and the likely presence on the ground (Fig. 1). Justifications for the inclusion of individual datasets can be found in Supplementary Information.

We identified 22 biodiversity feature datasets that are split into 54 separate triggers. Table 2 presents these datasets, with references pointing to where the data can be downloaded and notes denoting whether the data are new for this version or only available on request from data providers. Two datasets, the World Database of Key Biodiversity Areas and the IUCN Red List, are only available under a CC BY-NC licence on request from, respectively, the IUCN Red List GIS team (see https://www.iucnredlist.org/resources/spatial-data-download for data and https://www.iucnredlist.org/terms/terms-of-use indicating no commercial use) and BirdLife International, on behalf of the KBA Partnership (https://www.keybiodiversityareas.org/kba-data/request for data and https://www.keybiodiversityareas.org/termsofservice indicating no commercial use).

**Table 2:** Biodiversity features included in the analysis, their alignment with IFC PS6 Critical Habitat criteria and classification as ‘Likely’ or ‘Potential’ Critical Habitat.

Biodiversity features	Designation criterion / Trigger	IFC PS6 criteria					Classification
		1	2	3	4	5	
Cold seeps^33,34^			**P**		**L**	**L**	Likely/Potential
Cold water corals	Modelled occurrence^35–37^				**P**	**P**	Potential
	Observed occurrence^38^				**L**	**L**	Likely
Ever-wet tropical forest^†^^39^					**P**		Potential
Great Apes habitat*^40,41^		**L**					Likely
Hydrothermal vents^42^	Active		**L**			**L**	Likely
	Inactive				**P**		Potential
Important Bird and Biodiversity Areas^†^^43^	A1	**L**					Likely
	A2		**P**				Potential
	A3				**P**		Potential
	A4			**L**			Likely
	B1b	**P**					Potential
Important Marine Mammal Areas*^†^^44^	A	**P**					Potential
	B1		**P**				Potential
	B2			**L**			Likely
	C1, C2, and C3			**P**			Potential
	D1		**P**		**P**		Potential
	D2				**P**		Potential
Intact Forest Landscapes*^45,46^					**L**		Likely
Irreplaceable protected areas^†^^47^					**L**		
IUCN Red List of Threatened Species^†^^10^	CR under criterion D	**L**					Likely
	EN under criterion D	**L**					Likely
	VU under criterion D2	**P**					Potential
Key Biodiversity Areas^†^^48^	Alliance for Zero Extinction Sites	**L**	**L**	**L**			Likely
	A1a and A1e	**L**					Likely
	A1b	**P**					Potential
	A2a				**L**		Likely
	A2b				**P**		Potential
	B1		**L**				Likely
	B4				**P**		Potential
	C				**P**		Potential
	D1a			**L**			Likely
	D1b			**P**			Potential
	D2			**L**			Likely
	D3			**P**			Potential
	E	**P**					Potential
Mangroves^28,49^					**L**		Likely
Saltmarsh^50,51^					**L**		Likely
Sea turtle nesting sites^52,53^	All sea turtle species			**P**	**P**		Potential
	CR and EN sea turtle species	**L**					Likely
Seagrass beds^54^					**L**		Likely
Seamounts^55,56^					**P**		Potential
Tiger Conservation Landscapes^57,58^		**L**					Likely
Tropical dry forest^59,60^					**P**		Potential
Tropical moist forest*^29,61^					**L**		Likely
Tropical montane cloud forests^62,63^					**L**		Likely
Warm water coral reefs^64^					**L**	**L**	Likely
World Database on Protected Areas^9^	All Ramsar sites				**L**		Likely
	Ramsar sites under criterion 2	**L**					Likely
	Ramsar sites under criteria 5 and 6			**L**			Likely
	Ramsar sites under criteria 1 and 3				**L**		Likely
	Ramsar sites under criteria 4, 7, 8 and 9			**P**			Potential
	Protected areas under IUCN management categories Ia, Ib, and II				**L**		Likely
	Natural and mixed World Heritage Sites				**L**		Likely

*Data newly added in the update. ^†^Data only available on request from respective authors.

Note: CR: Critically Endangered; EN: Endangered; L: Likely; P: Potential. The majority of IBAs qualify as KBAs.

### Data processing and spatial analysis

Spatial data analysis was conducted in **R**, predominantly using the *terra* and *sf* packages.

For the greatest accuracy, vector data were processed using the *sf* package and *S2* library. This allows spatial processing, e.g. intersections and buffers, using Great Circle distances. Data were prepared for the analysis in three steps: First, data were made valid on the sphere. Second, data were filtered as detailed by (a) their respective guidance, and (b) to produce the data subset required for the analysis. For example, Key Biodiversity Areas (KBAs) must first be filtered by those whose status is confirmed (KbaStatus = “confirmed”) and then by the criterion/criteria required to create the subset (e.g. sites designated under KBA Criterion B1). Finally, data were unioned to remove duplicates from point data and any overlapping area in polygon data. Not all input data were available in vector format. For raster data, preprocessing varies by source but generally involved aggregating to the correct resolution (30 arcseconds) before reprojecting the data to a template raster in WGS 84.

Data are then converted to binary maps of presence/absence. For vector data, this was done by a process called rasterisation, whereby any grid cell in contact with the vector data assumes the value of the vector data. In keeping with the precautionary nature of the screening layer, we chose to rasterise based on any intersection, not requiring polygon data to cross the midpoint of the grid cell. Point data transferred their values to the grid cells they occupy. For raster data, where necessary (i.e. the data were not already binary), a classification threshold was set. Again, in keeping with the precautionary nature of the data, we set this at 0.5. This meant that any cell with >= 50% of the feature was classified as a presence (see Technical Validation for a brief sensitivity analysis of this threshold).

Binary raster data were then combined so that each grid cell’s unique combination of biodiversity features has a unique value. Based on what features comprised the value, the grid cell was categorised hierarchically: Likely > Potential > Unclassified.

## Data Records

The dataset is available through the UN Environment Programme World Conservation Monitoring Centre’s data portal (https://data-gis.unep-wcmc.org/portal/home/).

These data are presented in two formats at 30 arcseconds resolution in WGS 84 projection and one in WGS 84 vector format. They represent the best publicly available global screening layer for Critical Habitat:

**Basic Critical Habitat layer** ^25^

*Basic_Critical_Habitat_2025.tif*

Basic global layer at 30 arcseconds resolution in WGS 84 projection containing three values: 0 (Unclassified), 1 (Potential Critical Habitat), and 10 (Likely Critical Habitat). Made available under a CC BY licence at 10.34892/snwv-a025.

**Drill down Critical Habitat layer** ^26^

*Drill_Down_Critical_Habitat_2025.tif* and *Drill_Down_Critical_Habitat_2025.tif.vat.dbf*

More detailed global layer at 30 arcseconds resolution in WGS 84 projection. The spatial grid (*Drill_Down_Critical_Habitat_2025.tif*) works in conjunction with the raster attribute table (RAT: *Drill_Down_Critical_Habitat.tif.vat_2025.dbf*) to detail what biodiversity features trigger any cell’s Critical Habitat classification. Values in the TIFF file correspond to unique identifiers in the RAT that reveal the underlying data (Table 3). Made available under a CC BY-NC licence at 10.34892/d3xm-qm60.

**Table 3:** Codes for biodiversity features in the raster attribute table.

Shortcode	Description
VALUE	Unique identifier relating cells in the raster to data in this database
COUNT	Number of cells with this unique combination of features
CH	Critical Habitat classification
C1	Binary variable indicating IFC PS6 Criteria 1 triggered
C2	Binary variable indicating IFC PS6 Criteria 2 triggered
C3	Binary variable indicating IFC PS6 Criteria 3 triggered
C4	Binary variable indicating IFC PS6 Criteria 4 triggered
C5	Binary variable indicating IFC PS6 Criteria 5 triggered
L_AZE	Binary variable indicating presence of Alliance for Zero Extinction Sites
L_CLOUDFR	Binary variable indicating presence of Tropical montane cloud forests
L_COLDREEF	Binary variable indicating presence of Cold water coral reefs - Observed occurrence
L_COLDSEEP	Binary variable indicating presence of Cold seeps
L_CRCRD	Binary variable indicating presence of CR species under criterion D
L_ENCRD	Binary variable indicating presence of EN species under criterion D
L_GRTAPES	Binary variable indicating presence of Great Apes habitat
L_HYDRO	Binary variable indicating presence of Hydrothermal Vents
L_IBAA1	Binary variable indicating presence of IBAs under criterion A1
L_IBAA4	Binary variable indicating presence of IBAs under criterion A4
L_IFL	Binary variable indicating presence of Intact Forest Landscapes
L_IMMAB2	Binary variable indicating presence of IMMAs under criterion B2
L_IRREPL	Binary variable indicating presence of Irreplaceable protected areas
L_IUCNMGMT	Binary variable indicating presence of IUCN management categories Ia, Ib and II
L_KBAA1AE	Binary variable indicating presence of KBAs under criteria A1a and A1e
L_KBAB1	Binary variable indicating presence of KBAs under criterion B1
L_KBAD1A	Binary variable indicating presence of KBAs under criterion D1a
L_KBAD2	Binary variable indicating presence of KBAs under criterion D2
L_MANGROVE	Binary variable indicating presence of Mangroves
L_MOISTFOR	Binary variable indicating presence of Tropical moist forest
L_RAMS13	Binary variable indicating presence of Ramsar sites under criteria 1 and 3
L_RAMS2	Binary variable indicating presence of Ramsar sites under criterion 2
L_RAMS56	Binary variable indicating presence of Ramsar sites under criteria 5 and 6
L_RAMSALL	Binary variable indicating presence of All Ramsar sites
L_SALTM	Binary variable indicating presence of Saltmarshes
L_SEAGRSS	Binary variable indicating presence of Seagrass beds
L_TIGERCL	Binary variable indicating presence of Tiger Conservation Landscapes
L_TRTLCREN	Binary variable indicating presence of Sea turtle nesting sites - CR and EN species
L_WARMREEF	Binary variable indicating presence of Warm water coral reefs
L_WHS	Binary variable indicating presence of Natural and mixed World Heritage Sites
P_COLDREEF	Binary variable indicating presence of Cold water coral - Modelled occurrence
P_COLDSEEP	Binary variable indicating presence of Cold seeps
P_DRYFOR	Binary variable indicating presence of Tropical dry forest
P_EVERWET	Binary variable indicating presence of Ever-wet tropical forests
P_HYDRO	Binary variable indicating presence of Hydrothermal vents
P_IBAA2	Binary variable indicating presence of IBAs under criterion A2
P_IBAA3	Binary variable indicating presence of IBAs under criterion A3
P_IBAB1B	Binary variable indicating presence of IBAs under criterion B1b
P_IMMAA	Binary variable indicating presence of IMMAs under criterion A
P_IMMAB1	Binary variable indicating presence of IMMAs under criterion B1
P_IMMAC123	Binary variable indicating presence of IMMAs under criteria C1, C2 and C3
P_IMMAD1	Binary variable indicating presence of IMMAs under criterion D1
P_IMMAD2	Binary variable indicating presence of IMMAs under criterion D2
P_KBAA1B	Binary variable indicating presence of KBAs under criterion A1b
P_KBAB4	Binary variable indicating presence of KBAs under criterion B4
P_KBAD1B	Binary variable indicating presence of KBAs under criterion D1b
P_KBAD3	Binary variable indicating presence of KBAs under criterion D3
P_KBAE	Binary variable indicating presence of KBAs under criterion E
P_RAMS4789	Binary variable indicating presence of Ramsar sites under criteria 4, 7, 8 and 9
P_SEAMOUNT	Binary variable indicating presence of Seamounts
P_TRTLALL	Binary variable indicating presence of Sea turtle nesting sites - All species
P_VUCRD	Binary variable indicating presence of VU species under criterion D2

**Drill down Critical Habitat layer - polygons** ^26^

*Drill_Down_Critical_Habitat_Polygons_2025.gpkg*

This polygonised version of the drill down raster layer allows for more concise information retention (Table 4). The data draw boundaries around the cells of each unique feature combination. For ease, and to limit the size of the file, we excluded Unclassified polygons. There are 20,340 polygons in the GeoPackage. Made available under a CC BY-NC licence at 10.34892/d3xm-qm60.

**Table 4:** Variable names and descriptions in the polygonised GeoPackage layer.

Name	Description
CH	Critical Habitat designation: Likely, Potential, or Unclassified.
CRITERIA	IFC PS6 Criteria triggered.
ALL_FEATURES	All biodiversity features found in polygon.
C1	All biodiversity features triggering IFC PS6 Criteria 1.
C2	All biodiversity features triggering IFC PS6 Criteria 2.
C3	All biodiversity features triggering IFC PS6 Criteria 3.
C4	All biodiversity features triggering IFC PS6 Criteria 4.
C5	All biodiversity features triggering IFC PS6 Criteria 5.

## Data Overview

**Fig. 2:**
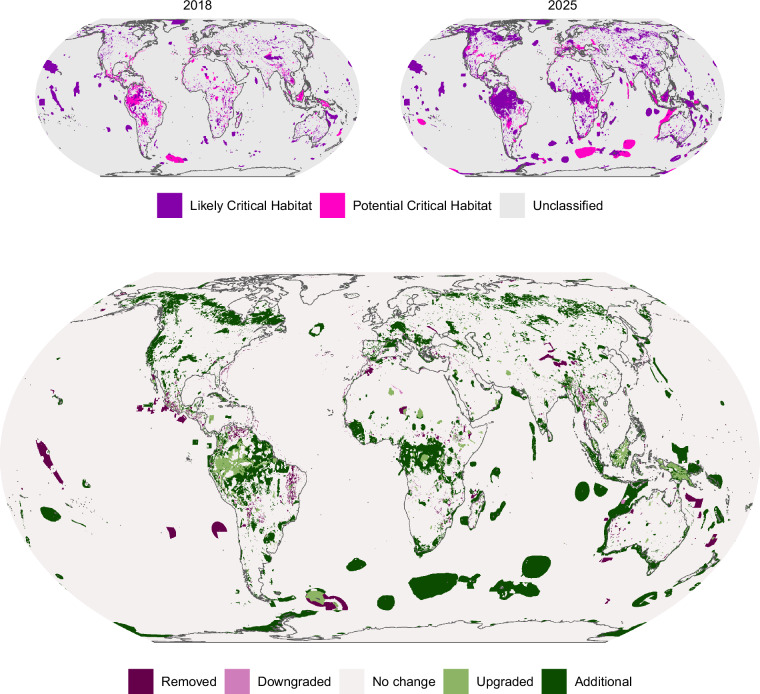
Global screening layer for Critical Habitat. Reprojected to Equal Earth and aggregated to 10 × 10 km for visualisation.

We find that 67.66 million km^2^ of the Earth’s surface was classified as Likely or Potential Critical Habitat (Fig. 2): 53.95 million km^2^ (10.58%) as Likely Critical Habitat and 13.71 million km^2^ (2.69%) as Potential Critical Habitat. This is a significant increase on the 25.78 million km^2^ (5.05%) and 10.1 million km^2^ (1.98%) previously identified. The remaining 442.4 million km^2^ (86.73%) is “Unclassified” as either known biodiversity features do not align with the IFC definition or because appropriate data that might be used to classify do not exist: Critical Habitat may still occur in these regions.

## Technical Validation

### Coverage

Both categories of Critical Habitat increased their absolute areas relative to the first global screening layer. Likely Critical Habitat maintained a largely similar split proportionally across domains (Table 5) but Potential Critical Habitat coverage increased in Areas Beyond National Jurisdiction (ABNJ) relative to land and Exclusive Economic Zones (EEZ). Overall, the updated screening layer increased coverage in all domains, with a large increase from 1.10% to 3.70% in ABNJ, 8.02% to 13.84% in EEZ and 15.09% to 27.22% on land.

**Table 5:** Areas and percentage coverage of each Critical Habitat category in 2018 and 2025 across realms.

	2018							2025					
	Likely		Potential		Unclassified			Likely		Potential		Unclassified	
ABNJ	2.4	9.2%	0.052	0.6%	220	46.4%		5	9.4%	3.2	23.2%	214	48.4%
EEZ	8.8	34.2%	2.472	24.4%	129	27.2%		14	26.8%	5.0	36.6%	121	27.4%
Land	14.6	56.6%	7.578	75.0%	125	26.4%		34	63.8%	5.5	40.4%	107	24.2%

Percentages indicate splits within Critical Habitat categories.

Note: Areas in km^2^ × 10^6^. ABNJ: Areas Beyond National Jurisdiction; EEZ: Economic Exclusion Zone

**Fig. 3:**
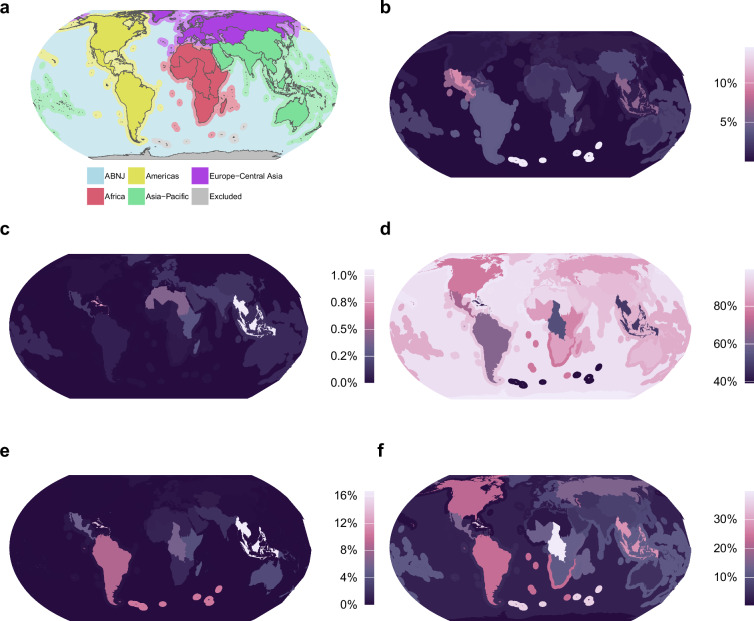
Regional trends in changes to the area of Likely or Potential Critical Habitat, reprojected to Equal Earth projection and shown as a percentage of each subregion’s area. Data uses subregions of the Intergovernmental Panel on Biodiversity and Ecosystem Services (IPBES) from Brooks *et al*.^65^. Land, Exclusive Economic Zones and Areas Beyond National Jurisdiction are calculated separately. Plots show (**a**) IPBES regions and subregions; (**b**) Likely or Potential Critical Habitat removed in this update; (**c**) downgraded; (**d**) no change; (**e**) upgraded; and (**f**) added. Note different scales.

Changes to the area of Potential or Likely Critical Habitat exhibit distinct regional patterns (Fig. 3). Subregions, as defined by the Intergovernmental Panel on Biodiversity and Ecosystem Services (IPBES), with the most changes to coverage are the Caribbean, Central Africa, South-East Asia, South America, and remote islands near or in the Southern Ocean (Fig. 3d). States (including associated territories) with the largest areas of Critical Habitat are shown in Table 6.

**Table 6:** States (including associated territories) with the largest areas of identified Critical Habitat.

State	Likely CH	Potential CH	Total
Areas Beyond National Jurisdiction	5,049	3,171	8,222
Australia	3,944	1236.7	5,181
United States of America (the)	4,213	893.2	5,106
Brazil	3,978	479.1	4,457
Russian Federation (the)	4,072	206.1	4,278
Canada	3,972	8.7	3,981
Indonesia	1,567	815.9	2,383
France	1,616	678.5	2,294
China	1,658	126.5	1,785
Congo (Democratic Republic of the)	1,473	77.4	1,551

Note: Areas in km^2^ × 10^6^.

Table 7 sets out the coverage of the Critical Habitat screening layer across the five criteria. Criteria 1, 3, and 4 dominate coverage. As identified previously^15,16^, data are still either largely unavailable or do not meet Criterion 5, key evolutionary processes, and all biodiversity features triggering it remain in the marine realm. The ongoing development and improved coverage of the IUCN Red List of Ecosystems will likely serve to harmonise much of the data inputs to Criterion 4 and reduce the percentage contribution of Criterion 4 to Likely and Potential Critical Habitat classifications^24,27^.

**Table 7:** Areas and percentage cover of each IFC PS6 criteria to Potential and Likely Critical Habitat.

Criteria	Likely		Potential	
1. Critically Endangered or Endangered species	28,020	52.0%	7,694	56.2%
2. Endemic and/or restricted-range species	14,486	26.8%	1,747	12.8%
3. Globally significant concentration of migratory or congregatory species	21,020	39.0%	6,994	51.0%
4. Highly threatened and/or unique ecosystems	38,931	72.2%	4,634	33.8%
5. Key evolutionary processes	521	1.0%	725	5.2%

Percentages indicate splits within Critical Habitat categories.

Note: Areas in km^2^ × 10^6^. Percentages add up to > 100 due to overlapping areas.

### Large species ranges

**Fig. 4:**
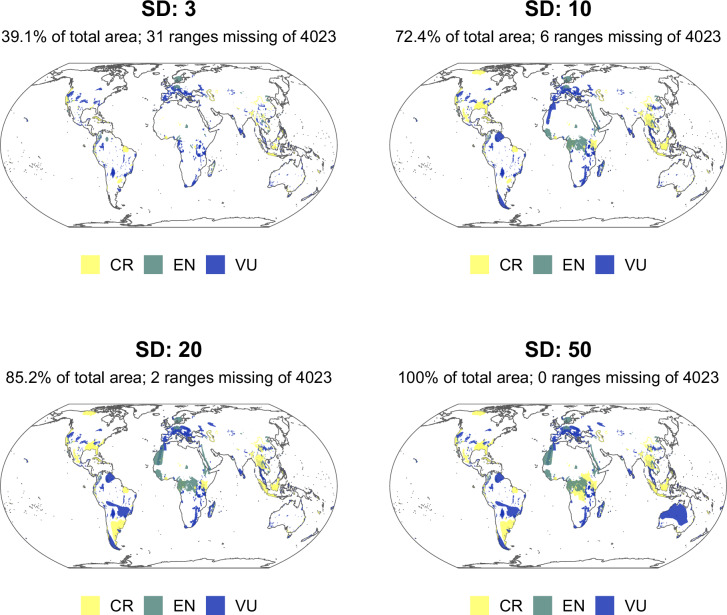
Sensitivity analysis of IUCN Red List ranges. Plots show result of removing areas with 3, 10, 20, 50 standard deviations (SDs) higher than the mean of the data. Each plot reports the SD, the number of ranges removed, and the percentage of the total area the data now represent.

The inclusion of species classified as Vulnerable in the IFC Guidance Note 2019 update, and data updates to the IUCN Red List generally, necessitated the inclusion of large numbers of species ranges. Some of these ranges, e.g. the Australian grey falcon, *Falco hypoleucos*, are exceptionally large. To retain precision in the updated screening layer, we conducted a sensitivity analysis of trimming the largest species ranges (range areas 3, 10, 20 and 50 standard deviations above the mean; see Fig. 4 below). Trimming species ranges 3 standard deviations above the mean resulted in a 61.2% decrease in coverage for the removal of only 31 species ranges out of 3,953. Furthermore, among the species ranges excluded were the northern white rhino, *Ceratotherium simum cottoni*, whose species range spans several Central and Eastern African countries despite comprising only two living individuals, and the ivory-billed woodpecker, *Campephilus principalis*, which may be extinct. Species are not expected to be evenly distributed within this range and if it is otherwise important for threatened species it will likely be classified as Critical Habitat via other biodiversity features.

### Presence thresholds

**Fig. 5:**
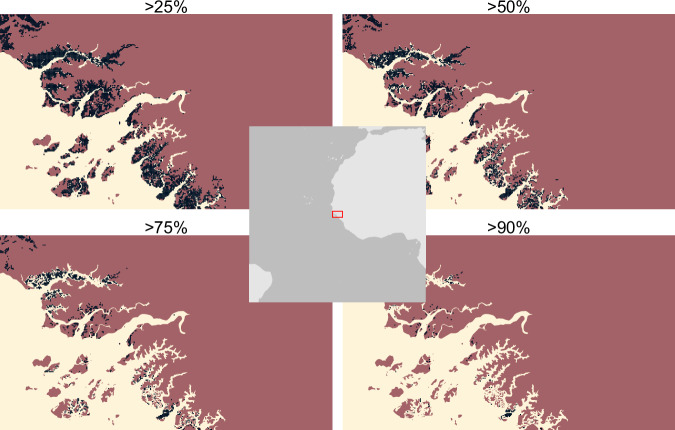
Sensitivity analysis of % presence cutoff values for mangrove data. Plots show effect of setting the % presence to >25, >50, >75, >90% on the output binary distribution. We select 50% for use in this analysis.

Following Martin *et al*.^15^ and Brauneder *et al*.^16^, we use a high threshold for species distribution models of >90% as for the modelled cold-water coral datasets used in this analysis (Table 2; see also Fig. 8). However, two datasets, tropical moist forest and mangroves, required an alternative approach as they represent high-resolution data (10 or 30 m grid cells) derived from satellite imagery^28,29^. The 10 or 30 m pixels are classified as presence/absence. When averaged to 30 arcseconds resolution, these data then present an effective percentage cover in the cell. Figure 5 below shows the result of applying four different thresholds to these data: 0.25, 0.5, 0.75, and 0.9. As the aggregated data do not constitute a probability distribution, we selected ≥ 0.5 as the threshold. For mangroves, the area occupied by 1 km cells over this threshold approximated the reported global extent of mangroves^28^.

### Feature coverage vs previous screening layer

**Fig. 6:**
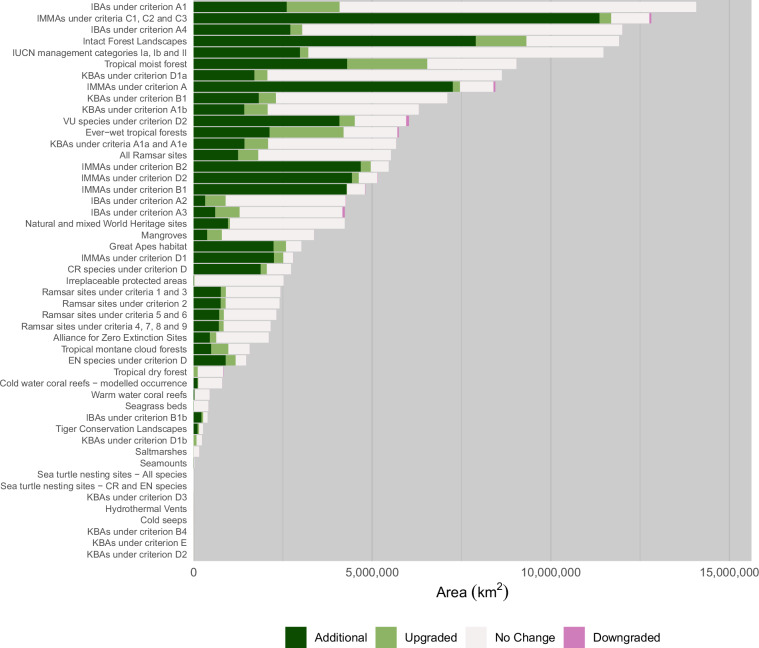
Critical Habitat (Likely and Potential) areas per biodiversity feature, split by whether the area is additional, upgraded, unchanged, or downgraded.

In our updated Critical Habitat screening layer, 38.03 million km^2^ has been added, 4.558 million km^2^ upgraded, 0.2585 million km^2^ downgraded, and 6.246 million km^2^ removed. This represents a significant expansion of area over previous efforts but likely remains an underestimation of actual Critical Habitat worldwide. This is due in part to the strict data screening criteria we set (Methods), but also a genuine lack of data for many biodiversity features. Tables 8, 9, 10 and 11 detail the biodiversity features responsible for the changes. Figure 6 shows the composition of each biodiversity feature.

**Table 8:** Biodiversity features contributing the five highest proportions to the area added as Potential or Likely Critical Habitat in the update.

Biodiversity feature	Area added	%	Total feature area	%
IMMAs under criteria C1, C2 and C3	11,367	29.89%	12,811	88.72%
Intact Forest Landscapes	7,900	20.77%	11,908	66.34%
IMMAs under criterion A	7,261	19.09%	8,447	85.97%
IMMAs under criterion B2	4,679	12.30%	5,461	85.67%
IMMAs under criterion D2	4,435	11.66%	5,159	85.98%

Note: Areas in km^2^ × 10^3^. IMMA: Important Marine Mammal Area. Percentages may add up to more than 100.

**Table 9:** Biodiversity features contributing the five highest proportions to the area upgraded from Potential to Likely Critical Habitat in the update.

Biodiversity feature	Area upgraded	%	Total feature area	%
Tropical moist forest	2,245	49.25%	9,037	24.84%
Ever-wet tropical forests	2,073	45.48%	5,743	36.10%
IBAs under criterion A1	1,480	32.47%	14,070	10.52%
Intact Forest Landscapes	1,419	31.13%	11,908	11.92%
IBAs under criterion A3	681.3	14.95%	4,232	16.01%

Note: Areas in km^2^ × 10^3^. Important Bird and Biodiversity Area. Percentages may add up to more than 100.

**Table 10:** Five largest areas of biodiversity features downgraded from Likely to Potential Critical Habitat in the update.

Biodiversity feature	Area downgraded	%	Previous feature area	%
KBA: 1 (CR/EN)	3,237	26.74%	69.12	2.13%
IBA: A3	5,048	25.02%	64.68	1.28%
KBA: 2a	1,547	21.38%	55.28	3.57%
Mangroves	487.2	12.88%	33.3	6.83%
Warm-water coral reefs	949.9	7.11%	18.37	1.93%

Names do not fully align with features in updated layer.

Note: Areas in km^2^ × 10^3^. CR: Critically Endangered; EN: Endangered; KBA: Key Biodiversity Area; IBA: Important Bird and Biodiversity Area. Percentages may add up to more than 100.

**Table 11:** Five largest areas of biodiversity features removed as Critical Habitat in the update.

Biodiversity feature	Area removed	%	Previous feature area	%
Tropical dry forest	2,380	16.65%	1,040	43.69%
PA (endemic/restricted range sp.)	3,647	15.02%	938.2	25.73%
KBA: 1 (CR/EN)	3,237	12.92%	806.8	24.92%
PA (CR/EN sp.)	3,761	12.74%	795.4	21.15%
Soft cold-water coral (Octocorals)	1,226	7.61%	475.6	38.81%

Names do not fully align with features in updated layer.

Note: Areas in km^2^ × 10^3^. CR: Critically Endangered; EN: Endangered; PA: protected area; KBA: Key Biodiversity Area. Percentages may add up to more than 100.

Important Marine Mammal Areas (IMMAs) dominate contributions to added Potential and Likely Critical Habitat (Table 8), adding 11.37 million km^2^ for IMMAs designated under criteria C1, C2 and C3 alone. Intact Forest Landscapes, an extensive biodiversity feature added in the update (see Supplementary Information for their inclusion justification), also contributes a significant area. For areas upgraded from Potential Critical Habitat to Likely Critical Habitat (Table 9), forest features dominate: over half of the area upgraded contain areas that are Likely Critical Habitat due to the presence of tropical moist forest. Just under half of all areas upgraded were previously Potential Critical Habitat due to the presence of tropical dry forest.

**Fig. 7:**
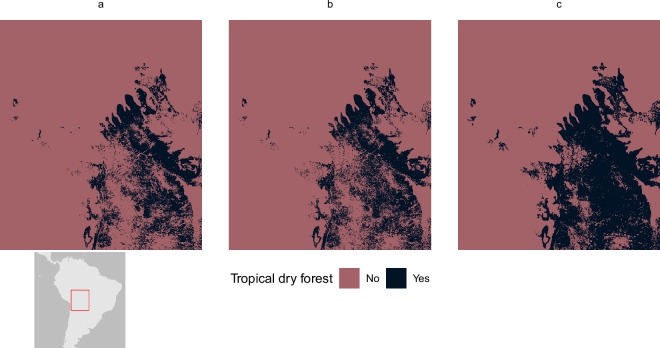
Differences in tropical dry forest coverage between (**a**) 2025 aggregation method, (**b**) original 500 m resolution, and (**c**) 2018 aggregation method.

**Fig. 8:**
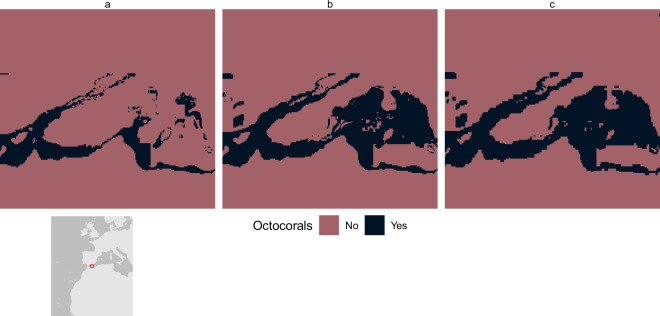
Differences in octocoral coverage between (**a**) 2025 threshold (>90%), (**b**) >  = 90% threshold, and (**c**) 2018 octocoral coverage.

Areas downgraded or removed from the screening layer tend to be an order of magnitude lower than those upgraded or added (Tables 10 and 11). Some of the features shown to have had area removed in the update are derived from the World Database on Protected Areas (WDPA) and World Database of Key Biodiversity Areas (WDKBA), two datasets that are regularly updated, with records both added and removed. Protected areas, for example, are often downgraded, downsized, or even degazetted and these changes would be reflected in the screening layer^30^. However, some features used the same data as previously, which warranted further inspection: 43.69% of tropical dry forest has been removed from the layer, alongside 38.81% of cold-water coral. Both appear to be small errors in the production of the previous screening layer. Figure 7 shows a comparison between the original input data, used in this layer and previously, alongside how the data were aggregated to a lower resolution in the previous layer and now. The method employed here appears to better reflect the higher resolution data but would reduce the overall feature area when compared to previously. For octocorals, the previous methodology considered values of >90% as presences for the species distribution models, but Fig. 8 shows that it is more likely that the analysis classified cells ≥90% as presences. In this update, we have maintained a threshold of >90%, which has consequently removed some areas from the layer.

## Usage Notes

As noted by Martin *et al*.^15^ and Brauneder *et al*.^16^, the global Critical Habitat screening layer does not replace detailed on-the-ground Critical Habitat assessments. Reference to Critical Habitat here refers specifically to areas in the screening layer identified as Likely or Potential Critical Habitat, and does not indicate confirmed, on-the-ground, Critical Habitat. The data allow users to identify areas that need further investigation, including full Critical Habitat assessment, and to help direct impact mitigation efforts and conservation action.

We recommend working mostly with the raster data. While the polygon layer allows for more information to be stored about the relevant biodiversity triggers, it is susceptible to misuse if users mistake it for actual vector data and start performing advanced spatial operations on the layer.

Due to precision restrictions inherent with **R**, the number of biodiversity feature triggers that this workflow can handle is ~66 (this analysis uses 54, see Table 2).

## Supplementary information

Supplementary Information

## Data Availability

All data described in this Data Descriptor are available on the UN Environment Programme World Conservation Monitoring Centre’s data portal (https://data-gis.unep-wcmc.org/portal/home/): • The basic layer^25^, *Basic_Critical_Habitat_2025.tif*, identifying 30 arcsecond grid cells that are either Likely Critical Habitat, Potential Critical Habitat or Unclassified, is available for download here: 10.34892/snwv-a025. • The drill down layer^26^, identifying both 30 arcsecond grid cells that are either Likely Critical Habitat, Potential Critical Habitat or Unclassified, as well as which biodiversity feature triggered any of the five criteria (Table 1), are available at 10.34892/d3xm-qm60 in two formats: ⚬ Raster (*Drill_Down_Critical_Habitat_2025.tif* and *Drill_Down_Critical_Habitat_2025.tif.vat.dbf*, see Table 3 for a description of the variables included); and ⚬ Polygon (*Drill_Down_Critical_Habitat_Polygons_2025.gpkg*, see Table 4). The data download, WCMC_043_GlobalCH_IFCPS6_2025, contains three subfolders and a short README file: 1. 01_Data: data files outlined above; 2. 02_Resources: relevant literature providing further information on IFC PS6; and 3. 04_Map: a simple global representation of the data in a .jpg plot. The basic layer is made available under a CC BY licence whereas the drill down layers are made available under a CC BY-NC licence.

## Code availability

The code used to compile this update of the global Critical Habitat screening layer is publicly available through the *Zenodo* repository^31^. The analysis is split across seven **R** scripts:

• *0 spatial_processing_functions.R*. Provides custom spatial processing functions required (see Methods).

• *1.1 Red_List_Preprocessing.R*. Initial processing of the IUCN Red List data, including filtering out species ranges with areas larger than a set amount of standard deviations above the mean (see Technical Validation).

•*1.2 Data_Preprocessing_Raster.R*. Preprocessing for the few datasets not available in vector format to produce standardised binary rasters for the next stage of the analysis.

•*1.3 Data_Preprocessing_Vector.R*. Preprocessing for vector input datasets to produce flat, dissolved layers for the next stage of the analysis.

• *2 Create_Drill_Down_Critical_Habitat_Raster_Layer.R*. Contains the lion’s share of processing. Converts the input flat vector layers to binary raster data and combines with the existing binary raster data in a process akin to the Combine tool in ArcGIS to produce a layer with a unique value for every resultant combination of input datasets. The script also produces the accompanying raster attribute table (RAT).

• *3 Create_Basic_Critical_Habitat_Raster_Layer.R*. Converts the detailed raster data into the basic Critical Habitat file.

• *4 Create_Drill_Down_Polygons.R*. Polygonises the drill down raster data to produce a vector layer that allows more information to be stored in the file than allowed in the spatial grid + RAT.
